# Regulation of the Bone Marrow Niche by Inflammation

**DOI:** 10.3389/fimmu.2020.01540

**Published:** 2020-07-21

**Authors:** Ioannis Mitroulis, Lydia Kalafati, Martin Bornhäuser, George Hajishengallis, Triantafyllos Chavakis

**Affiliations:** ^1^First Department of Internal Medicine, Department of Haematology and Laboratory of Molecular Hematology, Democritus University of Thrace, Alexandroupolis, Greece; ^2^National Center for Tumor Diseases (NCT), Partner Site Dresden, Germany and German Cancer Research Center (DKFZ), Heidelberg, Germany; ^3^Institute for Clinical Chemistry and Laboratory Medicine, University Hospital and Faculty of Medicine Carl Gustav Carus of TU Dresden, Dresden, Germany; ^4^Department of Internal Medicine I, University Hospital and Faculty of Medicine Carl Gustav Carus of TU Dresden, Dresden, Germany; ^5^Laboratory of Innate Immunity and Inflammation, Department of Basic and Translational Sciences, Penn Dental Medicine, University of Pennsylvania, Philadelphia, PA, United States

**Keywords:** hematopoietic stem cell, inflammation, niche, bone marrow, myelopoiesis

## Abstract

Hematopoietic stem cells (HSC) reside in the bone marrow (BM) within a specialized micro-environment, the HSC niche, which comprises several cellular constituents. These include cells of mesenchymal origin, endothelial cells and HSC progeny, such as megakaryocytes and macrophages. The BM niche and its cell populations ensure the functional preservation of HSCs. During infection or systemic inflammation, HSCs adapt to and respond directly to inflammatory stimuli, such as pathogen-derived signals and elicited cytokines, in a process termed emergency myelopoiesis, which includes HSC activation, expansion, and enhanced myeloid differentiation. The cell populations of the niche participate in the regulation of emergency myelopoiesis, in part through secretion of paracrine factors in response to pro-inflammatory stimuli, thereby indirectly affecting HSC function. Here, we review the crosstalk between HSCs and cell populations in the BM niche, specifically focusing on the adaptation of the HSC niche to inflammation and how this inflammatory adaptation may, in turn, regulate emergency myelopoiesis.

## Introduction: HSCs and Inflammation

**Figure 1:**
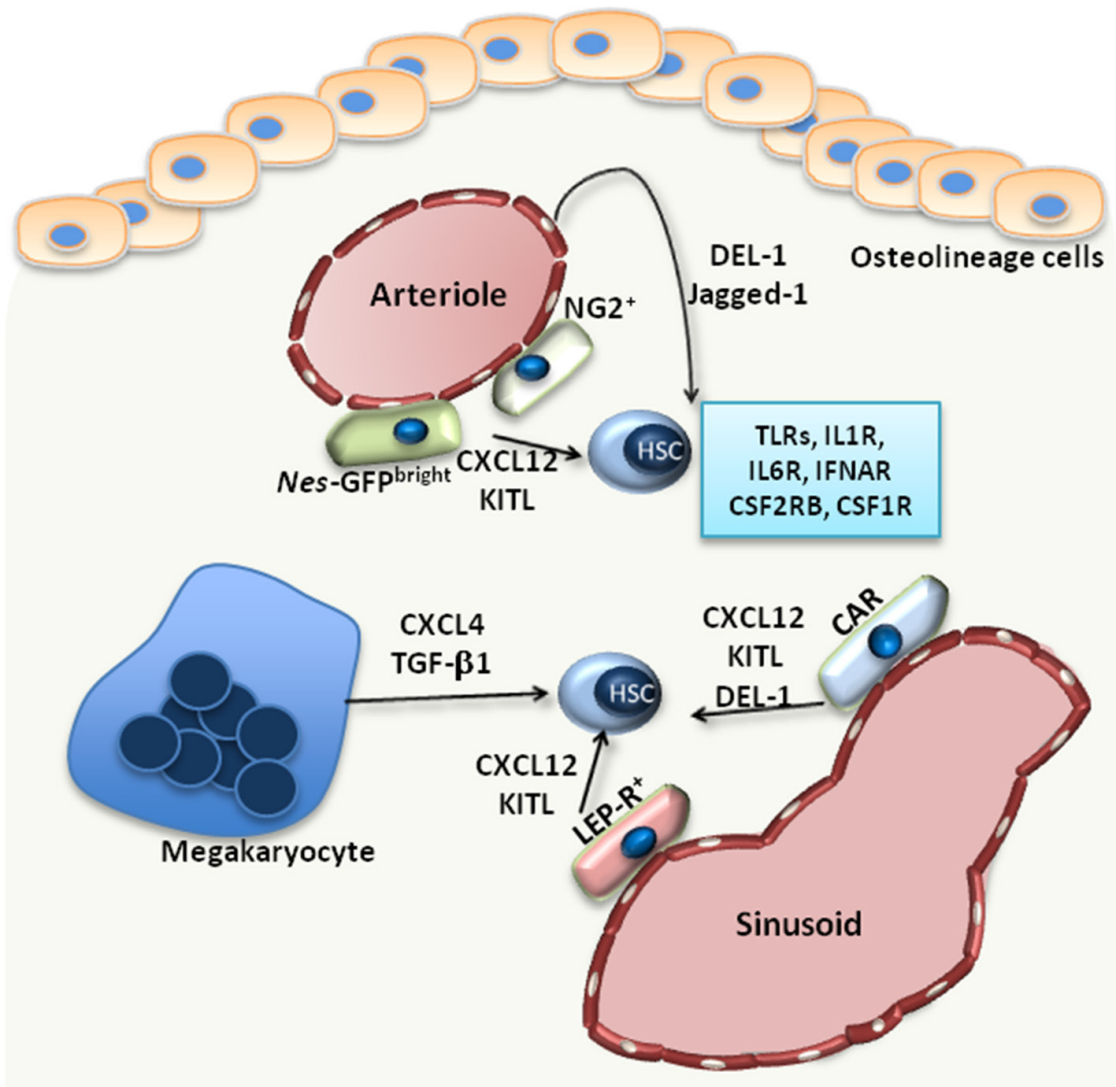
The HSC niche. Under steady state, HSCs reside in the proximity of BM vessels, either arterioles or sinusoids. Several MSC populations, including *Nes*-GFP^bright^, NG2^+^, LEP-R^+^ MSCs or CXCL12-abundant reticular (CAR) cells, promote the maintenance of HSCs by releasing factors such as CXCL12 or KITL. Endothelial cells also support HSCs via the release of Jagged-1 or DEL-1, a factor also produced by CAR cells. Megakaryocytes produce CXCL4 and TGF-β1, which also promote HSC maintenance in the BM. Additionally, HSCs are equipped with receptors, such as TLRs, interleukin-1 receptor (IL1R), interleukin-6 receptor (IL6R), IFN-α receptor (IFNAR), CSF1R, CSF2RB, that enable their direct response to inflammatory stimuli.

Hematopoietic stem cells (HSC) have self-renewal capacity and give rise to all mature blood cells, a process defined as hematopoiesis. The maintenance of the rare HSC population in the bone marrow (BM) and the preservation of their functional properties is supported by a highly specialized microenvironment inside the BM, the HSC niche (1, 2). Besides extracellular matrix, the HSC niche (Figure 1) comprises different cell populations, including mesenchymal stromal cells (MSC), endothelial cells, osteolineage cells as well as progeny of HSCs, such as macrophages and megakaryocytes (1–3). The niche supports HSCs either *via* direct adhesive interactions (between HSCs and the cells or the extracellular matrix of the niche) or through the secretion of factors that act in a paracrine manner. Such factors with paracrine effects on HSCs include C-X-C motif chemokine ligand (CXCL)-12, thrombopoietin, transforming growth factor (TGF)-β1 or stem cell factor (SCF; also called Kit-ligand) (1, 2). The fine regulation of HSCs by the BM niche microenvironment promotes maintenance of HSC quiescence, which is critical for preservation of their self-renewal potential (1).

Hematopoietic stress drives HSCs to exit their quiescent state and undergo proliferation and lineage differentiation, according to the demands of the specific hematopoietic stress. In the case of systemic inflammation or infection, the resulting stress on hematopoiesis creates a tremendous need for production of mature myeloid cells, especially neutrophils and monocytes (3, 4). This vital and pressing response of the BM to infections or peripheral inflammation is termed emergency myelopoiesis (5). Hematopoietic stem and progenitor cells (HSPCs) are fully equipped with pattern-recognition receptors (6), e.g., Toll-like receptors (TLR), and respond to pathogen-derived products that reach the BM through the circulation. TLR activation of HSPCs leads to enhanced proliferation and myeloid cell production (6–8), thereby promoting mature myeloid cell replenishment in the course of infection (6). HSCs can also respond to several growth factors and cytokines released during inflammation by immune or other cells at inflamed sites or by cells within the BM microenvironment. For instance, interleukin (IL)-1 or IL-6 act directly on HSCs, driving their proliferation and instructing their differentiation toward the myeloid lineage (9–11), while type I interferon [e.g., IFN-α induces the proliferation of HSCs (12). However, chronic exposure to cytokines may injure HSCs (9, 12, 13)]. Moreover, HSCs express the receptor for macrophage colony-stimulating factor (CSF1; also known as M-CSF), CSF1R, and respond to *in vitro* M-CSF stimulation with differentiation toward monocytes (14). The inflammatory adaptation of HSPCs can also contribute to induction of trained immunity, i.e., a non-specific memory of previous encounters that promotes enhanced responses by HSPCs and their progeny to future challenges (3, 15). Specifically, agonists of trained immunity may stimulate the upregulation of the expression of the common β subunit of the granulocyte macrophage colony–stimulating factor/interleukin-3 receptor (CSF2RB), thereby promoting downstream signaling and leading to enhanced myelopoiesis (10). The direct effects of pathogen-derived factors, cytokines and growth factors on HSPCs have been recently reviewed elsewhere (3).

In the context of inflammatory stress, not only HSCs, but also cell populations that form the HSC niche, sense and respond to inflammatory stimuli, such as pathogen-derived products, cytokines or growth factors; this response is crucial for ensuring the steady replenishment of leukocytes (3–5). The focus of the present review is the adaptation of the HSC niche cells to inflammation. Specifically, we review here the role of HSC niche inflammation in emergency myelopoiesis and in the context of malignant hematopoiesis.

## HSC Niche at Steady-State

Under steady-state conditions, the function of the BM niche is to regulate the maintenance of HSCs. Imaging studies revealed that HSCs localize in the perivascular space; however, there are discrepant reports with regards to the localization of HSCs at the endosteal area around arterioles (endosteal niche) (16) or around sinusoids (vascular niche) (17). Perivascular cells of mesenchymal origin are main cellular niche components (Figure 1) and hence critical regulators of HSC maintenance (1). The identification of such mesenchymal cell populations relies on genetically modified mice serving as reporters for different markers, including Nestin (NES), Kit ligand (KITL), CXCL12, nerve/glial antigen 2 (NG2) and Leptin receptor (LEP-R) (16, 18–20). Using *Nes*-GFP reporter mice, Kunisaki et al. identified two distinct cell populations of *Nes*-GFP-positive MSC with different transcriptional profiles; namely, *Nes*-GFP^bright^ cells, which have a peri-arteriolar localization (Figure 1), and *Nes*-GFP^dim^ cells, which are rather in the proximity of sinusoids (16). Furthermore, NG2+ pericytes were found spatially linked to arterioles, in intimate contact with HSCs, thereby supporting the quiescence of the latter (16) (Figure 1). Another study by Acar et al. demonstrated that HSCs are localized in close contact with LEP-R^+^ and CXCL12^high^ cells in the peri-sinusoidal area, rather than the peri-arteriolar area (17) (Figure 1). HSC function can be modulated by the aforementioned perivascular cells, as demonstrated by conditional deletion experiments. Deletion of CXCL12 or KITL from all pericytes, characterized as *Nes*-GFP+ cells, resulted in depletion of HSCs (21). CXCL12 deletion in arteriolar NG2^+^ cells decreased numbers of HSCs and affected their spatial localization in the BM, whereas deletion of CXCL12 in sinusoidal LEP-R^+^ cells had no effect (21). In contrast, KITL released by LEP-R^+^ sinusoidal cells, but not by NG2^+^ arteriolar cells, was important for HSC preservation (21).

Besides perivascular mesenchymal cells, endothelial cells are also important regulators of HSC function. Arterial rather than sinusoidal endothelial cells are an important source of KITL in the BM (22). Moreover, arteriolar endothelial cells together with CXCL12-abundant reticular cells also release developmental endothelial locus (DEL)-1 in the HSC niche (23) (Figure 1). DEL-1 is a glycoprotein that supports HSC proliferation and myeloid lineage instruction through interactions with β3 integrin expressed on HSCs (23, 24). The angiocrine factor Jagged-1 is another endothelial cell-derived factor that promotes HSC maintenance in a Notch-dependent manner and hematopoiesis in the context of regeneration (25) (Figure 1).

Recent advances in single-cell technologies enabled the characterization of HSC niche populations at the single-cell level. Single-cell transcriptomics enabled the identification of two endothelial, four perivascular, and three osteolineage cell populations with distinct transcriptional profiles (26). In the same study, arteriolar perivascular cluster was found to express *Cxcl12, Kitl*, whereas sinusoidal LEP-R^+^ cluster expressed, besides *Cxcl12* and *Kitl, Il-7, Il-15*, and *Csf1* (encoding M-CSF) (26).

Megakaryocytes also represent a niche component contributing to the maintenance of HSCs in the BM. HSCs are localized in proximity to megakaryocytes, and depletion of megakaryocyte resulted in a significant decrease of HSC numbers (27), as megakaryocyte-derived CXCL4 promotes HSC quiescence in the BM (27). Another study confirmed the spatial association between megakaryocytes and HSCs and demonstrated that megakaryocytes are an important source of TGF-β1 in the BM, which also promotes HSC quiescence (28) (Figure 1). Macrophages also exert functions in the HSC niche. CD169^+^ macrophages interact with MSCs in the niche, thereby regulating the expression of important retention molecules, such as CXCL12 (29). Furthermore, macrophages expressing α-smooth muscle actin are localized in proximity to HSCs and support the release of CXCL12 by stromal cells (30). The circadian regulation of CXCL12 expression by stromal cells, which in turn influences the release of HSCs from the BM, is under the control of sympathetic nerve fibers (31).

## HSC Niche Adaptation To Inflammation

**Figure 2:**
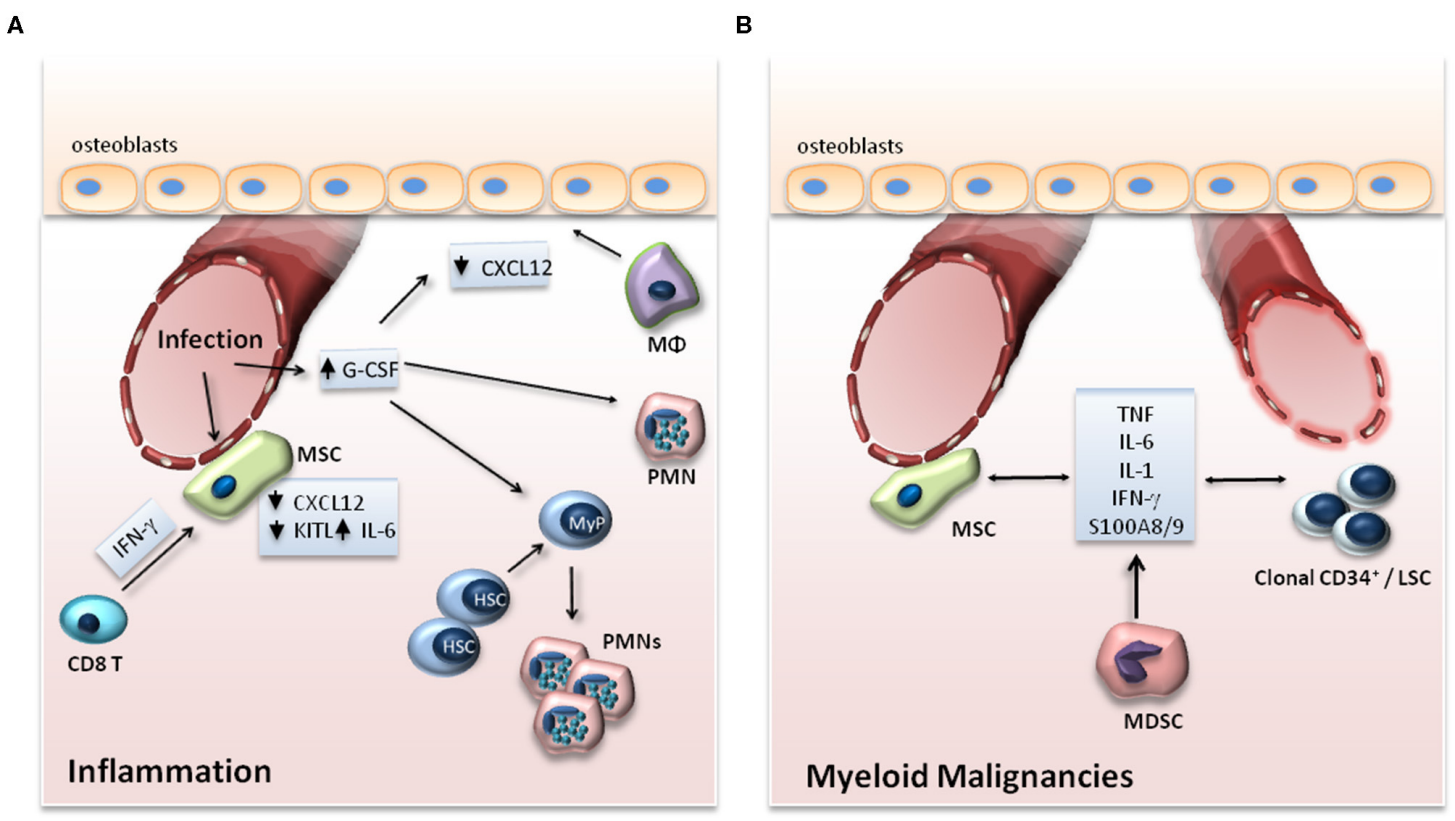
**(A)** Adaptation of the HSC niche to inflammation. Upon infection, expression of G-CSF in endothelial cells and of IL-6 in endothelial cells and MSCs is increased, while the expression of CXCL12 and KITL, which support the maintenance of HSCs in the BM, is downregulated. G-CSF drives myeloid differentiation of progenitor cells and suppresses the expression of CXCL12 by osteoblasts either directly or through functional changes in macrophages (Mφ) or granulocytes. In viral infections, IFN-γ produced by CD8+ T cells acts on MSCs in the BM niche leading to enhanced release of IL-6 by the MSCs. **(B)** BM niche in myeloid malignancies. In myeloid malignancies, clonal CD34^+^ progenitor cells alter their microenvironment through the production of inflammatory mediators, which may affect the vasculature in the endosteal area of the BM and promote an inflammatory signature in stromal cell populations. The production of cytokines by MSCs as well as by myeloid-derived suppressor cells (MDSCs) may also support clonal expansion of clonal CD34^+^ cells and leukemic stem cells (LSCs).

Niche cell populations may regulate the hematopoietic response to peripheral inflammation or infection, for instance, by releasing factors that promote myelopoiesis (5, 32, 33) (Figure 2A). Such a factor is granulocyte colony-stimulating factor (G-CSF), a central regulator of infection-induced emergency granulopoiesis, as it exerts a key role in the differentiation of progenitors of the myeloid lineage to mature granulocytes (5). Endothelial cells, rather than cells of hematopoietic origin, have been demonstrated as the main source of G-CSF in the BM niche during inflammation (33, 34). In the course of LPS-induced systemic inflammation, TLR4 signaling in endothelial cells was responsible for increased G-CSF production and consequent emergency granulopoiesis (33).

Although there are contradictory reports regarding the ability of G-CSF to induce cell-cycle entry in HSCs (35–37), G-CSF is well-established as a factor mediating the mobilization of HSCs. In fact, G-CSF is clinically engaged for inducing HSC mobilization to peripheral blood in the context of therapeutic transplantation (38). Moreover, G-CSF contributes to HSC mobilization during infection. Indeed, systemic infection of mice with *Escherichia coli* induces HSC mobilization from the BM and their accumulation in the spleen (39), in a manner dependent on TLR4-induced production of G-CSF and on signaling via nucleotide-binding oligomerization domain (NOD)-containing proteins; however, TLR4 expressed on HSCs was not required (39). To promote mobilization of HSCs, G-CSF acts on different cells in the BM niche, including MSCs, osteolineage cells, neutrophils and macrophages, (40). Importantly, administration of G-CSF reduces the expression of the retention molecules, such as *Cxcl12, Spp1* (osteopontin), *Kitl, Angpt1* (angiopoietin 1), and *Vcam1* (vascular cell adhesion molecule 1) in BM MSCs (41) and *Cxcl12* in osteolineage cells (42). G-CSF can directly disrupt osteoblast activity and downregulate the expression of *Ccxl12* in these cells (42). Additionally, G-CSF can affect the levels of CXCL12 in the endosteal niche through indirect mechanisms; namely, through the depletion of endosteal macrophages (43), the release of granulocyte-derived proteases (44) or through signals from sympathetic nervous system that may suppress osteoblast function (45).

Mature leukocytes can also influence BM microenvironment during infection, for instance, by releasing reactive oxygen species (ROS) (46). ROS, released in the extracellular space, act in a paracrine manner to induce the proliferation of myeloid progenitors during pathogen-induced emergency myelopoiesis (46). Furthermore, in the context of BM transplantation, tumor necrosis factor (TNF) derived from granulocytes acts on bone marrow endothelial cells promoting vessel and hematopoietic regeneration (47). Specifically, adoptive transfer of granulocytes supported the recovery of sinusoids and the reconstitution of hematopoiesis after transplantation in a TNF-dependent manner. The effect on vascular regeneration was lost in recipient mice deficient for TNF receptors, suggesting a direct action of neutrophil-derived TNF on endothelial cells. Moreover, TNF acted directly on hematopoietic progenitors, since the effect of granulocyte transfer was decreased in mice that were transplanted with hematopoietic cells from TNF receptor-deficient mice (47).

IFNs may also modulate HSC niche populations during inflammation. In a model of lymphocytic choriomeningitis virus (LCMV) infection, IFN-γ indirectly enhances myelopoiesis by acting on MSCs (48). Specifically, IFN-γ secreted by cytotoxic CD8+ T cells activates MSCs and stimulates the expression of IL-6 in the latter, which in turn enhances myelopoiesis during the viral infection (48). Another study demonstrated that treatment of mice with IFN-α or polyinosinic:polycytidylic acid (polyI:C) activates BM endothelial cells and increases vascularity and vessel permeability (49). Moreover, single-cell transcriptomic analysis revealed that the administration of polyI:C and LPS induces gene expression of the chemokines *Ccl5, Ccl6, Ccl19, Cxcl9, Cxcl10*, and *Cxcl11* in CXCL12-abundant reticular cells and sinusoidal endothelial cells (32). Additionally, inflammation induced the expression of *Il6* and downregulated the expression of factors that contribute to HSC retention and lymphopoiesis, including *Cxcl12, Kitl*, and *Il7* (32).

Adhesive interactions within the HSC niche also contribute to the adaptation of hematopoiesis during inflammation. The αvβ3 integrin is implicated in the effect of IFN-γ on the suppression of HSC function (50). Engagement and signaling through β3 integrin in HSCs enhances STAT-1 phosphorylation and STAT-1 dependent gene expression upon IFN-γ administration to mice, leading to impaired repopulation potential of HSCs (50). Furthermore, the β3 integrin on HSCs mediates the regulatory effect of DEL-1 in myelopoiesis (23). DEL-1 expression in the BM niche was observed in endothelial cells and CXCL12-abundant reticular cells. The interaction of DEL-1 with β3 integrin promotes HSC proliferation and myeloid priming and thereby supports the regeneration of both steady-state and emergency myelopoiesis in response to LPS-induced systemic inflammation (23). Specifically, mice deficient in DEL-1 have decreased numbers of myeloid cells in the BM under steady state and a delayed recovery of myeloid cell populations after BM transplantation or upon LPS administration (23). Osteopontin is a ligand for β1 integrins on HSCs (51) and may also regulate myelopoiesis during inflammation. In a model of sterile inflammation and during acute fungal infection with *Candida albicans*, osteopontin acts on progenitor cells and suppresses emergency myelopoiesis (52). Taken together, inflammation alters the function of different cell populations in the HSC niche, whereas factors deriving from niche cells modulate the response of HSPCs during inflammation-induced emergency myelopoiesis.

## Inflammation In The HSC Niche During Malignant Hematopoiesis

Myeloid malignancies, such as myelodysplastic syndrome (MDS), acute myeloid leukemia (AML), and myeloproliferative neoplasms (MPN), represent clonal diseases of HSCs (53). Malignant clones bear mutations in regulators of major cell functions, such as epigenetic modifiers (*TET2, ASXL1, EZH2, DNMT3A, IDH*), pre-mRNA splicing machinery (*SF3B1, SRSF2, U2AF1*), transcription factors (*RUNX1, NPM1, ETV6, GATA2*), tumor suppressors (*TP53*), cohesion complex proteins (*STAG2*), tyrosine kinases (*FLT3, JAK2, MPL*) and signaling intermediates thereof (*RAS, CBL*) (53). Emerging evidence indicates that inflammation in the BM may contribute to the development and progression of myeloid malignancies (54) (Figure 2B).

Analysis of a population-based registry revealed that chronic inflammatory stimulation is a potential triggering factor for MDS and AML development (55). Changes in both number and function of different immune cell populations are observed in MDS patients. Inflammatory mediators, including S100A8/A9 or cytokines, such as TNF, IL-1β, IL-6, and IFN-γ, are increased in MDS (56). BM-derived MSCs from MDS patients display an inflammatory signature as well as upregulation of molecules involved in cell adhesion and angiogenic factors (57, 58). Activation of the nuclear factor kappa B (NF-κB) transcription factor program was also observed in MSCs of patients with MDS (59). Of interest, S100A8/A9, secreted in the BM by both MSCs and cells of hematopoietic origin, has been associated with activation of the inflammasome and consequent production of IL-1β and IL-18 by hematopoietic progenitors in MDS (56, 58). Moreover, S100A8/9 released by MSCs from patients with MDS induces DNA damage to hematopoietic progenitors, leading to cell-cycle arrest, cell death and progression toward leukemia (58). Also contributing to the disease phenotype is the ability of S100A9 signaling to induce ROS production, inflammasome activation and pyroptosis in hematopoietic progenitors from patients with MDS (60). Another study further identified S100A9 to act on myeloid-derived suppressor cells (an innate immune cell type with immunosuppressive potential enriched in the BM of MDS patients) and to upregulate secretion of suppressive factors (e.g., TGF-β and IL-10), thereby indirectly facilitating the expansion of the malignant clone (61). Intriguingly, IL-1β levels in the BM and IL-1 receptor expression in CD34^+^ progenitor cells are higher in AML patients. *Ex vivo* studies with leukemic stem cells identified IL-1β as a factor that drives expansion of myeloid progenitors from AML patients and suppresses the proliferation of normal CD34^+^ progenitors (62). In MPN, leukemic myeloid cells can affect and remodel the HSC niche in a way that offers advantages to leukemic stem cells rather than to healthy stem cells (63). Decreased numbers of BM MSCs were observed in patients and mice bearing the activating mutation *JAK2(V617F)*, due to a decrease in sympathetic nerve fibers, which support MSC abundance and function (64). Mutations leading to activation of the protein tyrosine phosphatase SHP2 (encoded by the PTPN11 gene) are linked to a specific MPN form, juvenile myelomonocytic leukemia. Hematopoietic cell-intrinsic effects of the aforementioned mutations are integral to MPN pathogenesis (65). Intriguingly, however, activating Ptpn11 mutations in cellular niche components, such as MSCs and osteoprogenitors, may also contribute to MPN development (66). Specifically, mutations in niche cells result in the increased production of the chemokine CCL3, leading to monocyte recruitment to the HSC niche, which in turn produce inflammatory cytokines, such as IL-1β, which can drive myeloproliferation (66). Furthermore, in a thrombopoietin (TPO)-dependent mouse model of myelofibrosis, differentiation of Gli1^+^ MSCs toward myofibroblasts results in BM fibrosis; consistently, Gli1^+^ MSCs numbers are increased in patients with MPN (67). Transcriptomic analysis revealed that these cells acquire an inflammatory signature during myelofibrosis, suggesting a link between inflammation and fibrosis in MPN (67).

Studies in mouse models point to clonal hematopoietic cells and their progeny as a substantial cellular source of factors contributing to BM inflammation and progression of clonal hematologic pathologies. Hematopoietic progenitor cells from mice deficient for Tet methylcytosine dioxygenase *2* (TET2), a gene frequently mutated in patients with myeloid malignancies, show enhanced self-renewal potential and an increased myeloid lineage bias (68). Additionally, HSCs from TET2-deficient mice show a different response to inflammatory stimuli compared to normal HSCs (69). Specifically, in stark contrast to normal HSCs whose self-renewal capacity is compromised by inflammation, TET2-deficient HSCs maintain their self-renewal capacity and are resistant to apoptosis upon LPS-induced systemic inflammation, a combination that promotes their expansion (69). *In vitro* treatment of hematopoietic progenitor and mature myeloid cells with LPS revealed increased expression of several pro-inflammatory cytokines, including IL-6, IL-1, and TNF, owing to TET2 deficiency (69). IL-6, in turn, mediates the enhanced pro-survival and proliferative response of *Tet2*^−/–^ progenitor cells to inflammation through hyperactivation of a Shp2-Stat3–dependent axis (69). As alluded to above, *Tet2*^−/–^ progenitors thrive under inflammatory conditions *in vitro* at the expense of Tet2-sufficient progenitors, thus *Tet2*^−/−^ clones appear to have increased fitness over normal clones in an inflammatory environment (69–71). Another study further supported the critical role of inflammation in the progression of clonal hematologic disease. Specifically, IL-6 produced in response to microbial infection results in pre-leukemic myeloproliferation in *Tet2*-deficient mice (72). Taken together, clonal hematopoietic cells often display increased pro-inflammatory potential that promotes inflammation within the BM. This, in turn, confers a competitive advantage for preferential proliferation of clonal hematopoietic cells, hence generating a vicious cycle between inflammation and malignant myelopoiesis.

The interplay between niche inflammation and myeloproliferation has been exemplified in further studies. In an acute myeloid leukemia mouse model, mice with constitutively activating internal tandem duplication (ITDs) of FLT3 (FLT3-ITD) show enhanced myeloproliferation and progressive loss of HSC function (73). The functional impairment of HSCs in FLT3-ITD mice was cell extrinsic and depended on the inflammatory modulation of the vascular niche populations (73). Specifically, *Tnf* expression was upregulated in BM endothelial cells. Consistently, blockade of TNF signaling with the TNF inhibitor etanercept partially rescued HSC dysfunction (73). Another study in the MLL-AF9-driven mouse AML model has shown that leukemia cell-expressed factors, specifically TNF and CXCL2, mediate the remodeling of endosteal vessels, generating a BM niche that supports preferentially leukemic clones resulting in the decrease of normal HSCs (74).

## Conclusion

Under steady-state conditions, the crosstalk between cellular components of the niche and HSCs enables the maintenance of the latter in the BM in a state of quiescence, thereby ensuring the preservation of HSCs (2). During inflammation, HSCs are activated, proliferate, and preferentially differentiate toward the myeloid lineage (3). In parallel to the direct effect of inflammatory stimuli on HSCs, inflammatory alterations in niche cell populations and signals thereof also regulate the adaptation of HSCs to emergency myelopoiesis (3) (Figure 2A). In addition, the interplay amongst HSCs, niche cell populations and inflammation is of critical importance in myeloid malignancies (54). The release of inflammatory mediators by clonal hematopoietic populations remodels the HSC niche in a manner that favors the preferential expansion of clonal leukemic cells, hence promoting the emergence and progression of malignant myeloid disease (54) (Figure 2B). Therefore, it is imperative that future investigations focus on better understanding of how inflammation regulates the interactions of HSCs with their niche both during normal and malignancy-associated hematopoiesis.

## Author Contributions

All authors listed have made a substantial, direct and intellectual contribution to the work, and approved it for publication.

## Conflict of Interest

The authors declare that the research was conducted in the absence of any commercial or financial relationships that could be construed as a potential conflict of interest.
